# Latent factors underlying the symptoms of adult-onset myotonic dystrophy type 1 during the clinical course

**DOI:** 10.1186/s13023-024-03359-8

**Published:** 2024-11-01

**Authors:** Yanan Zhang, Bailey Wallace, Bo Cai, Nicholas Johnson, Emma Ciafaloni, Yedatore Swamy Venkatesh, Christina Westfield, Suzanne McDermott

**Affiliations:** 1 Department of Epidemiology and Biostatistics, Arnold School of Public Health, University of South Carolina, Columbia, SC USA; 2 Division of Birth Defects and Infant Disorders, National Center on Birth Defects and Developmental Disabilities, Centers for Disease Control and Prevention, Atlanta, GA USA; 3 Oak Ridge Institute for Science and Education, Atlanta, GA USA; 4 Department of Neurology, Virginia Commonwealth University, Richmond, VA USA; 5 Department of Neurology, University of Rochester, Rochester, NY USA; 6 South Carilina Department of Public Health, Columbia, SC USA; 7 Department of Environmental, Occupational, Geospatial Health Sciences, Graduate School of Public Health and Health Policy, City University of New York, New York, NY USA; 8 Department of Neurology, School of Medicine Columbia, University of South Carolina, Columbia, SC USA

**Keywords:** Myotonic dystrophy, Neuromuscular diseases, Signs and symptoms, Factor analysis, Correlation, Public health surveillance

## Abstract

**Background:**

Myotonic dystrophy type 1 (DM1) is a multisystem genetic disorder that classically presents with symptoms associated with myotonia, early onset cataracts, and muscular weakness, although the presentation and pattern of disease progression is quite varied. Presenting symptoms are well documented among adults with DM1. However, less is known about the co-occurrence of symptoms over time. We aimed to use factor analysis to explore the correlation pattern of signs and symptoms (S/S) that emerged during the clinical course.

**Results:**

Clinical records of 228 individuals with adult onset DM1 were abstracted using the Muscular Dystrophy Surveillance, Tracking, and Research Network (MD STAR*net*) from a six-site cohort in the United States during an eight-year study period. Factor analysis was used to group the correlated S/S into latent factors. Three factors were identified. Group 1: ‘Facial Weakness/Myotonia’ includes the two most common S/S, as indicated by its name. Group 2: ‘Skeletal Muscle Weakness’ includes eight muscular S/S and is more frequently reported by males and those with older age at onset. Group 3: ‘Gastrointestinal distress/Sleepiness’ includes four non-muscular S/S and hand stiffness. The abstracted medical records reported that over 63% of individuals had S/S from all three groups. Associations of covariates with factor scores were also examined using linear regression. CTG repeat length was significantly positively associated with higher factor scores for all three factors.

**Conclusions:**

This study identified three latent factors of S/S which accumulated during the clinical course of adult onset DM1.

**Supplementary Information:**

The online version contains supplementary material available at 10.1186/s13023-024-03359-8.

## Background

Myotonic Dystrophy Type 1 (DM1) is an autosomal dominant genetic disorder. It is the most frequent form of muscular dystrophy, with historical prevalence estimates of 5–20 cases per 100,000 population [1–4] and higher prevalence estimates in certain parts of the world with founder populations, including the Saguenay-Lac-St-Jean area in Canada with a prevalence of 158 per 100,000 people [5]. DM1 has been reported to be the most common muscular dystrophy among adults of European ancestry, and it is rare among nonwhite populations [6–8]. A recent U.S.-based study using newborn bloodspots reported the genetic prevalence of the underlying CTG repeat length in the dystrophia myotonic-protein kinase (DMPK) gene, which is associated with DM1, was approximately one in 2,100 births although this estimate includes both asymptomatic cases and those that do not necessarily survive into adulthood [9].

DM1 is a degenerative disorder that affects multiple organ systems such as muscular, cardiovascular, digestive, and nervous systems. DM1 classically presents with symptoms associated with myotonia (e.g., delayed muscle relaxation) and muscular weakness, but symptoms are highly variable across individuals. Additionally, symptomology varies by sex; males are reported more likely to have cognitive impairment, myotonia, and cardiac and respiratory symptoms while females are more frequently reported to have signs and symptoms (S/S), such as cataracts, obesity, and gastrointestinal problems [10]. Except in those with congenital onset and in the most severe cases, the diversity of S/S in terms of age of onset, timing of clinical manifestations, severity, and progression hinder early diagnosis [11]. The mean age of genetic testing excluding the congenital cases for a definitive diagnosis is 33.4 years with a standard deviation (SD) of 12.5 years, after an average of approximately 7 years of seeking a diagnosis [12].

A higher CTG repeat length is associated with an earlier age of onset and greater severity for DM1 patients [13, 14]. Based on the age of onset and severity, DM1 is classified as: congenital DM1 (onset at birth and CTG repeat length typically greater than 1000); childhood-onset DM1 (occurring between age 1 to 10 years old and CTG repeat length is typically greater than 800); and adult-onset DM1 (onset later than 10 years old and CTG repeat length can vary from 50 to 1000). The adult-onset category includes the phenotype of classic DM1 (age of onset: 10–30 years), and phenotype of late onset/asymptomatic DM1(age of onset: 20–70 years) [15–17].

Declines in muscle strength, particularly distal muscles, fatigue and daytime sleepiness, cognitive functioning, and daily and social living over time are well documented among adults with DM1 [18–21]. However less is known about the co-occurrence of symptoms over the clinical course for people with DM1. Factor analysis has been used to explore the correlation patterns of S/S, allowing for the discovery of symptoms that tend to co-occur [22–24]. These correlated symptoms form clusters, often termed latent factors, indicating the presence of an underlying causal mechanism shared by those grouped symptoms.

Our objective was to identify the potential latent factors among S/S that develop during the clinical course. This can help health care providers and patients anticipate the progression of DM1 after diagnosis. Our study further examined the association between CTG length and sex with the identified S/S factors.

## Methods

### Population studied and data source

This is a cross-sectional study utilizing data from the Muscular Dystrophy Surveillance, Tracking, and Research Network (MD STAR*net*), funded by the Centers for Disease Control and Prevention (CDC), a network that conducts population-based surveillance, longitudinal follow-up, and public health research about muscular dystrophies. The individuals identified during the surveillance period (January 1, 2008- December 31, 2016) had clinical records of varying lengths; some individuals were followed for a few visits, and others had many years of data available (see Table 1a, 1b). The analysis focuses on symptoms of each patient reported up until their last available medical provider visit.

**Table 1a:** Categorical characteristics by sex, among adult-onset DM1 participants from MD STAR*net* (*n* = 228)

Categorical Characteristics	All		Female		Male		Tests
	*n*	%	*n*	%	*n*	%	
Clinical review status							𝜒2 = 0.81, DF = 1, *p* = 0.37
Definite	179	78.5	97	80.8	82	75.9	
Probable	49	21.5	23	19.2	26	24.1	
Race/ethnicity							𝜒2 = 2.82, DF = 1, *p* = 0.09
Non-Hispanic White	164	71.9	92	76.7	72	66.7	
Other ^a^	64	28.1	28	23.3	36	33.3	
Family History							𝜒2 = 0.82, DF = 2, *p* = 0.78
Yes	195	85.5	102	85.0	93	86.1	
No	29	12.7	15	12.5	14	13.0	
Not available	4	1.75	3	2.50	1	0.93	
Sites							𝜒2 = 2.50, DF = 5, *p* = 0.78
Colorado	38	16.7	17	14.2	21	19.4	
Iowa	35	15.4	21	17.5	14	13.0	
North Carolina Piedmont Region	49	21.5	26	21.7	23	21.3	
Western New York	45	19.7	26	21.7	19	17.6	
South Carolina	26	11.4	13	10.8	13	12.0	
Utah	35	15.4	17	14.2	18	16.7	
CTG repeat length							𝜒2 = 4.62, DF = 4, *p* = 0.33
[50–150)	29	12.7	13	10.8	16	14.8	
[150, 500)	60	26.3	35	29.2	25	23.1	
[500, 1000)	55	24.1	33	27.5	22	20.4	
1000 and plus	16	7.0	6	5.0	10	9.3	
Not available ^b^	68	29.8	33	27.5	35	32.4	

*Note*^a^: Other includes Hispanic, non-Hispanic across multiple races (American Indian or Alaska Native, Asian, Black, multiple, and 5 unknown race), and 35 unknown ethnicity group. ^b^: 68 individuals lacked a laboratory report for CTG repeat length, however, their myotonic dystrophy status was confirmed either genetically from a family member or supported by family history

**Table 1b:** Continuous characteristics by sex, among adult-onset DM1 participants from MD STAR*net*

Continuous Characteristics	Statistics	All	Female	Male	Tests
CTG repeat length	n	160	87	73	Wilcoxon rank sum test *p* = 0.48
	median (p25, p75)	426 (180,800)	446 (200,850)	400 (150,800)	
Age of onset	n	170	93	77	t = -1.00, DF = 168, *p* = 0.32
	mean ± SD	34.3 ± 15.1	33.3 ± 14.3	35.6 ± 15.9	
Age at genetic test	n	160	88	72	t = -0.62, DF = 158, *p* = 0.53
	mean ± SD	40.3 ± 15.4	39.6 ± 14.3	41.1 ± 16.7	
Diagnostic delay from onset to genetic test ^a^	n	114	67	47	t = 0.84, DF = 112, *p* = 0.40
	mean ± SD	6.5 ± 7.3	6.9 ± 7.7	5.8 ± 6.7	
Number of clinical visits with S/S reported	n	228	120	108	t = 0.67, DF = 226, *p* = 0.50
	mean ± SD	3.6 ± 1.9	3.6 ± 2.1	3.5 ± 1.7	
Years of follow-up since onset	n	170	93	77	t = 1.91, DF = 167, *p* = 0.06
	mean ± SD	9.5 ± 8.1	10.5 ± 8.9	8.2 ± 6.8	

*Note*^a^: 119 patients have age at S/S and age at genetic test. 114 patients’ age of onset is younger than age at genetic test, and 5 patients whose age at onset is greater than age at genetic test. SD = standard deviation

The surveillance sites were the U.S. states of Colorado, Iowa, 31 counties in North Carolina’s Piedmont region, South Carolina, Utah, and a 21-county area in Western New-York. Eligible individuals in these catchment areas were residents who had a clinic visit for DM1 during the eight-year study period. Earlier versions of the MD STAR*net* methodology are described in detail elsewhere [25, 26]. Here we briefly describe the methodology for the data used in this analysis.

MD STAR*net* used multi-source case finding with a focus on neuromuscular clinics. Other data sources included hospitals and hospital discharge databases, private physician practices, service sites for children with special health care needs, and birth defects surveillance programs. Identified individuals were linked to birth and death certificate data for more complete ascertainment of deaths. Potential DM1 individuals were identified by International Classification of Diseases, Ninth or Tenth Revision, Clinical Modification (ICD-9-CM or ICD-10-CM) codes (359.21, myotonic muscular dystrophy; G71.1, myotonic dystrophy; G71.0, muscular dystrophy) in the electronic medical records of specialty clinics, as well as death records.

Trained abstractors who were blinded to the study hypotheses screened records for demographic and clinical information. Two to three abstractors per MD STAR*net* site were trained in clinical aspects of DM and each site had a supervisor who did clinical review of the abstracted records. A MD STAR*net* database was used to code detailed diagnostic information, including S/S, initial history, physical examination, diagnostic notes, clinical progress notes, family history, electromyography reports (EMG), muscle biopsies, and genetic laboratory reports. Additional information, without a field, was copied verbatim from the clinical record into the database. Following data collection, a committee of seven neuromuscular clinicians, one neurologist or neuromuscular specialist per MD STAR*net* site, reviewed the abstracted diagnostic data to assign each individual a case status [27]. A definite case has myotonic dystrophy related clinical symptoms and genetically confirmed diagnosis by a clinical blood test (19q13.3 CTG repeat length > 50) either from the patient or from the family member. A probable case has related clinical symptoms and supported by family history of first-degree family member (either maternal or paternal transmission) consistent with DM1. Asymptomatic cases had a confirmed DNA diagnosis (same as Definite), but none of the following clinical symptoms: for children: weakness, myotonia (clinical or electrical); for adults: development of cataracts before age 50, myotonia (clinical or electrical), daytime hypersomnolence, or distal or proximal weakness.

### Study sample inclusion criteria

**Fig. 1:**
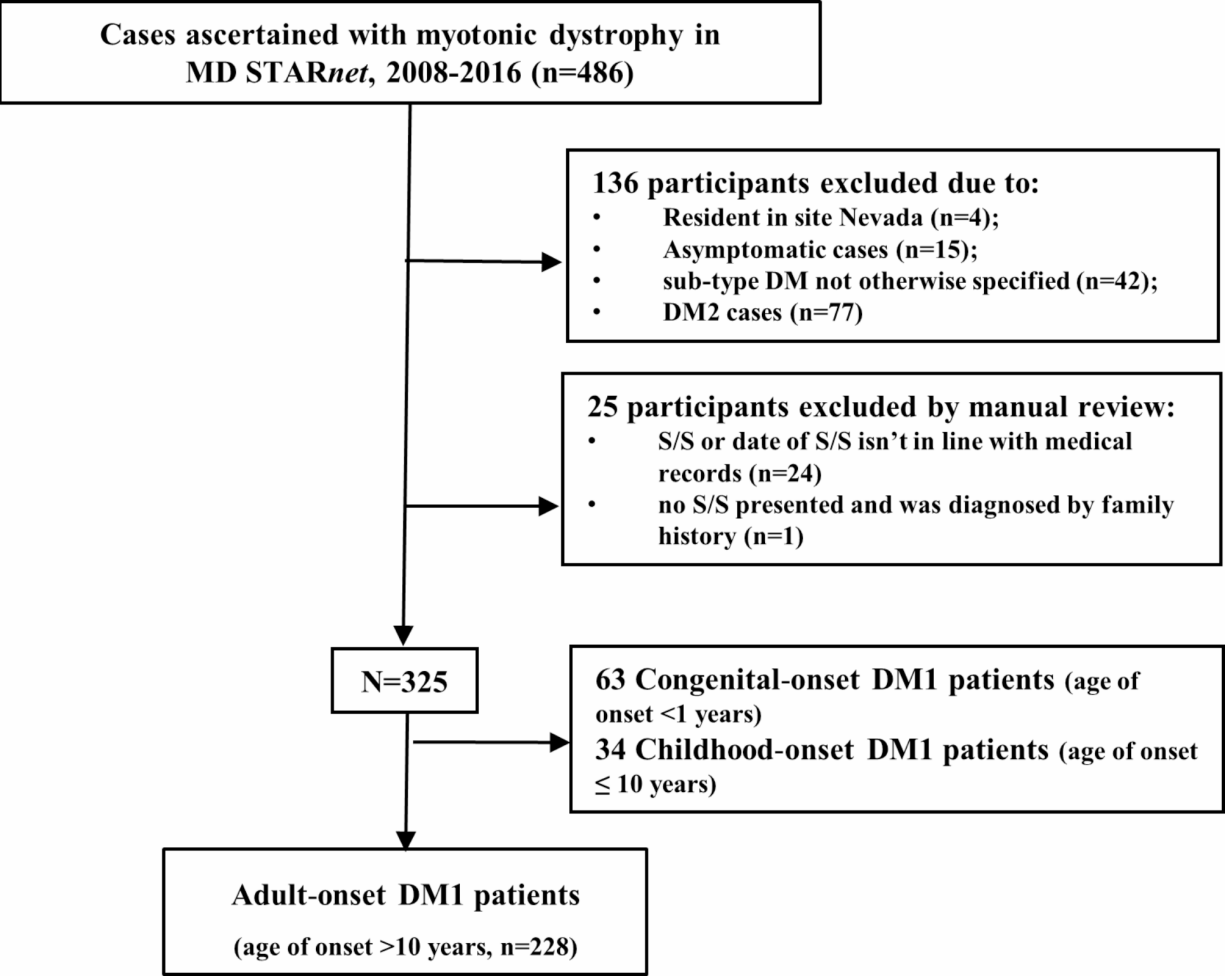
Flow chart of identified cases among adult-onset DM1 participants from MD STAR*net*. Based on the inclusion and exclusion criteria, a total of 228 individuals with adult-onset DM1 were included in the final analysis

Inclusion and exclusion criteria for this study are displayed in Fig. 1. There were 486 individuals ascertained with myotonic dystrophy in MD STAR*net* during the period 2008–2016. Considering the differences in gene mutations, age of onset, as well as clinical presentations, we included only individuals diagnosed with DM1 and with a definite or probable case status. We also excluded four individuals who resided in Nevada due to concerns of inconsistent case collection; 24 individuals because their S/S are not consistent with medical records, and one individual who had no record of S/S. We chose to study adult-onset DM1 since three quarters of people with DM1 do not report S/S until they are older than ten years, and congenital and early childhood DM1 have different features in their presentation and a different trajectory of disease progression [16]. The final study sample was 228 individuals with onset after age 10 years.

### Outcomes

The S/S in all clinical records were extracted, beginning with disease history, symptoms recorded during the initial contact with a neurologist or neuromuscular clinic, and S/S identified throughout subsequent visits. After grouping synonyms (Supplementary Table 2), twenty-one core clinical S/S were identified. While symptoms may be identified in multiple medical records, they are not analyzed as repeated measures. Regardless of when the symptoms were presented, the surveillance methodology only allowed for recording of symptoms until the patient’s last available medical provider visit.

### Predictors /covariates

We considered two primary independent variables: sex (male and female) and CTG repeat length (number of repeats of a CTG triplet in the 3’ non-coding region of DMPK). We classified CTG repeat length into the following categories: [(50–150), (150–500), (500–1000), and ≥ 1000]. For individuals who didn’t have genetic test results, their CTG repeat length was coded as ‘Not available’. We also considered two potential confounders, race/ethnicity and family history. Due to small case counts in some categories, we combined race and ethnicity into non-Hispanic White and other (including Hispanic, non-Hispanic across multiple races, and unknown ethnicity). Based on the family history in the medical records, family history was categorized into ‘Yes’, ‘No’ and ‘Not available’. Age of onset was defined as the age of the earliest reported S/S. Age at genetic test was defined as the age when the first genetic test was done. The length of follow-up was measured from the date of onset to the last available date of medical provider’s visit.

### Statistical analysis

Categorical characteristics were described by their frequencies and percentages, and their differences were assessed by 𝜒2 or Fishers Exact test for small cell sizes. The continuous characteristics were described by mean and standard deviation or median and interquartile range (IQR), and their differences were tested by t-test or Wilcoxon rank sum test when normality was violated.

We first used exploratory factor analysis (EFA) to group the 19 S/S listed in Table 2. S/S reported by less than 10 individuals (respiratory failure/insufficiency, developmental delays) were considered rare and were not included in the factor analysis (pairwise deletion). Considering the convention that requires 10–15 cases per item, and with 19 items (S/S) in our study, the sample size (*n* = 228) was sufficiently powered for EFA. We estimated the polychoric correlation matrix, and we applied oblimin rotation by assuming factors are correlated since S/S are rarely partitioned into neatly independent factors. Weighted least squares adjusted to mean and variance was used as the estimator. The number of factors was decided by the combination of scree plot and model fitting statistics (Supplementary Table 1). A cutoff point of 0.3 was used to decide which S/S were included in a factor [28]. Myalgia had loadings less than 0.3 and were removed, and the EFA was refit. For S/S with cross-loadings greater than 0.3, we classified the S/S into the factor with the higher loading.

**Table 2:** Number of adult-onset DM1 individuals with each sign and symptom, from MD STAR*net* (*N* = 228)

21 Signs and Symptoms ^a^	*n*	%
Myotonia	180	78.9
Facial Weakness/Trouble Smiling	146	64.0
Distal Weakness	142	62.3
Hand Weakness	136	59.6
Proximal Weakness	100	43.9
Trouble Walking / Running / Jumping	99	43.4
Ptosis	98	43.0
Dysarthria	95	41.7
Hand Stiffness	90	39.5
Cataracts Before 50 (for all individuals, *n* = 228) ^b^	89	39.0
Dysphagia	89	39.0
Chronic Fatigue	84	36.8
Daytime Sleepiness ^c^	81	35.5
Gastrointestinal Distress ^c^	77	33.8
Sleep Apnea	52	22.8
Myalgia ^c^	46	20.2
Trouble Climbing Up & Down Stairs	34	14.9
Jaw Stiffness	31	13.6
Cognitive Impairment	15	6.6
Respiratory Failure / Insufficiency	< 11	NR
Developmental Delays ^c^	< 11	NR

*Note*^a^: the number of patients reporting a symptom is not mutually exclusive. ^b^: Among 69 individuals who are ≥ 50 years old, 31 (44.9%) reported having Cataracts Before 50 years old. ^c^: This term includes a range of signs and symptoms, please refer to the Supplementary Table 2 for detailed information. In factor analysis, only signs and symptoms with over 10 patients were included. NR = no report for signs and symptoms among less than 11 patients

We then conducted a confirmatory factor analysis (CFA). Three statistically non-significant S/S (jaw stiffness *p* = 0.688, cognitive impairment *p* = 0.332, and cataracts before 50 for those over the age of 50 years *p* = 0.101) were excluded from further analysis. Individuals were assigned a factor score, which was computed by a count of S/Ss and their loadings. The associations of factor scores (outcome) with the logarithm of CTG repeats, age of onset, sex, race/ethnicity, family history status, years of follow-up since onset, and MD STAR*net* surveillance site were explored by fitting a linear regression model. Given the sample size and dimension of symptoms, we fit EFA and CFA using the same dataset, which could result in overfitting.

We assumed the missing data were non-informative for age of onset (*n* = 58, 25%), CTG repeat length (*n* = 68, 30%) and family history (*n* = 4, 1.8%). The sample size reduced from 228 to 115 when fitting the linear regression. All tests were two-tailed, and type I error probability was set at 0.05. For multiple linear regression, the false discovery rate was controlled using the Benjamini-Hochberg procedure. Specifically, we fit three separate multiple linear regressions examining the significance of six covariates, resulting in a total of 18 tests. The Benjamini-Hochberg procedure was applied by ranking the 18 p-values in ascending order. Each p-value was compared to a threshold of 0.05*rank/18 (e.g., the smallest to 0.05/18, the second smallest to 0.05*2/18, the next smallest to 0.05*3/18, and so forth, up to the largest p-value compared to 0.05). EFA and CFA were done using Mplus and R software. Descriptive statistics and linear regressions were done using SAS software 9.4 (SAS Institute Inc). All analyses were replicated by a second analyst.

## Results

This analysis includes 228 individuals from six geographic areas in the US, composed of individuals who were 72% non-Hispanic white and 13% reported no family history. Among the 160 individuals whose genetic information was available, the median CTG repeat length was 426 (IQR:180–800), and the mean diagnostic delay was 6.5 years (SD: 7 years) from the age of onset (mean ± SD: 34 ± 15 years) to genetic test (mean ± SD: 40 ± 15 years). The mean length of follow-up from disease onset to the last available medical visits is 9.5 years, and the average number of clinical visits with S/S reported is 3.6. There were no statistically significant differences by sex for any of the characteristics (Table 1a. & 1b).

The average number of S/S per patient was 7.4. Myotonia, facial weakness/trouble smiling, distal weakness, and hand weakness were found in over half of the individuals. Cataracts before age 50 years was present in 45% of the people older than 50 years. Other S/S, such as cognitive impairment, developmental delays, and respiratory insufficiency, were present in a small proportion of the cases (Table 2).

**Fig. 2:**
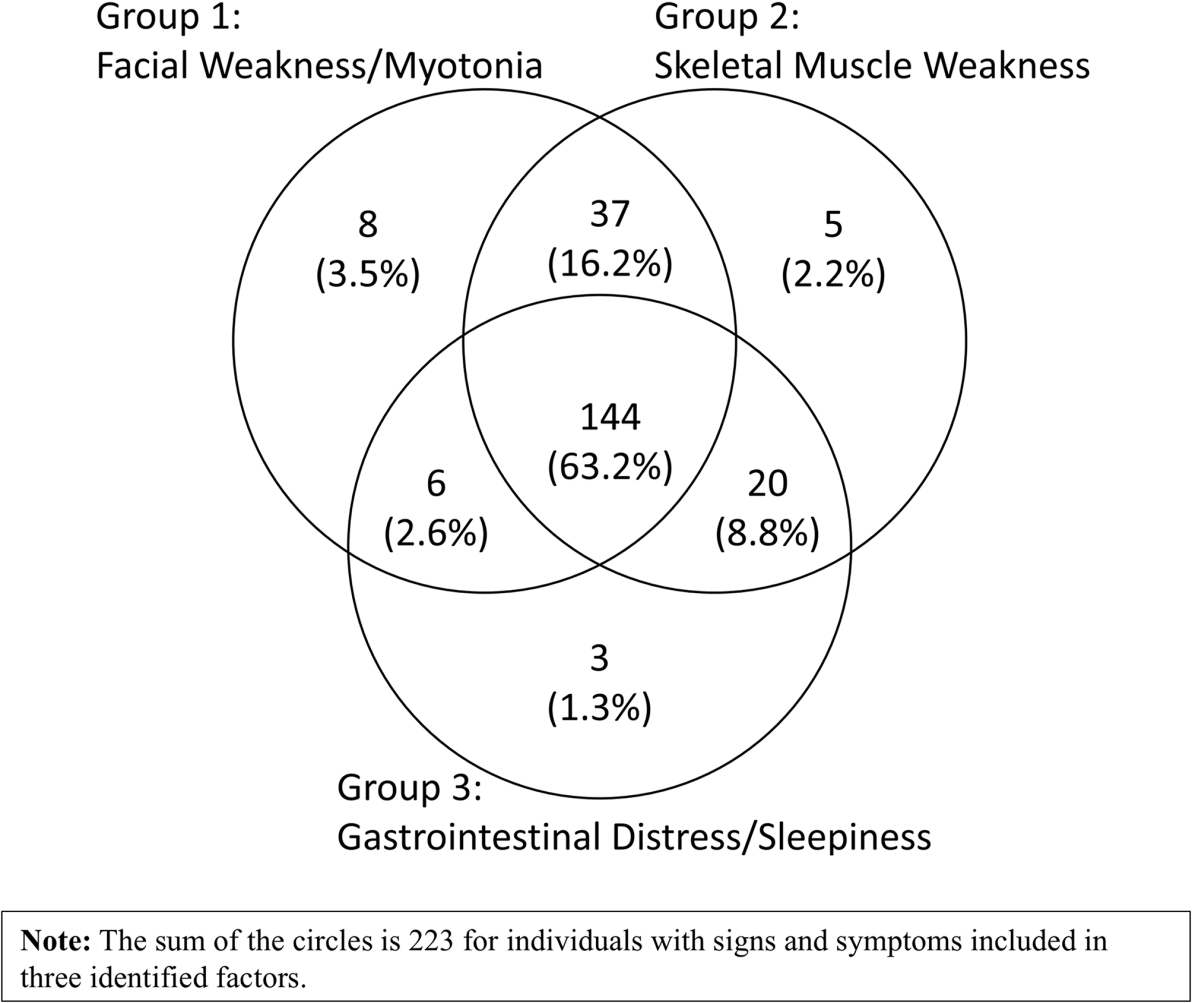
Venn diagram of signs and symptoms among adult-onset DM1 participants from MD STAR*net* (*N* = 228). 63.2% of the individuals reported all three groups of symptoms during the life course with DM1; 16.2% had S/S from Group 1 and 2; 8.8% from Groups 2 and 3; and 2.6% from Groups 1 and 3. In addition, there were five individuals who had cataracts as their only S/S. The overlap of the groups of signs and symptoms highlights the multidimensional nature of adult-onset DM1

Three latent factors were identified from the factor analysis (Table 3). Group 1 (‘Facial Weakness/Myotonia’) includes the two most recognized presentations, myotonia (78.9%, Table 2) and facial weakness/trouble smiling (64%, Table 2). Group 2 (‘Skeletal Muscle Weakness’) includes eight S/S that are mainly muscular related. Group 3 (‘Gastrointestinal Distress/ Sleepiness’) includes four non-muscular symptoms (Gastrointestinal Distress, Daytime Sleepiness, Chronic Fatigue, Sleep Apnea) and hand stiffness (Table 3). The three groups of S/S are not exclusive. More than half (63.2%) of individuals reported all three groups of symptoms during the clinical course with DM1; 16.2% had S/S from Group 1 and 2; and 8.8% had S/S from Groups 2 and 3. Only five individuals had cataracts as their only S/S (Fig. 2).

**Table 3:** Three identified factors using signs and symptoms among adult-onset DM1 participants from MD STAR*net* (*N* = 228)

Groups (Factors)	Signs and Symptoms	EFA	CFA			Included in Factors
		Loading	Estimates	SE	*p* value	
Facial Weakness/Myotonia	Facial Weakness/Trouble Smiling	0.58	0.80	0.11	< 0.001*	Yes
	Myotonia	0.51	0.72	0.11	< 0.001*	Yes
	Cognitive Impairment	0.53	excluded due to insignificant CFA			No
	Jaw Stiffness	0.33	same as above			No
	Cataracts Before 50	-0.36	same as above			No
Skeletal Muscle Weakness	Distal Weakness	0.69	0.69	0.08	< 0.001*	Yes
	Ptosis	0.66	0.63	0.07	< 0.001*	Yes
	Trouble Walking/Running/Jumping	0.57	0.51	0.08	< 0.001*	Yes
	Proximal Weakness	0.54	0.49	0.08	< 0.001*	Yes
	Trouble Climbing Up & Down Stairs	0.51	0.44	0.11	< 0.001*	Yes
	Dysarthria	0.49	0.63	0.08	< 0.001*	Yes
	Hand Weakness	0.43	0.48	0.09	< 0.001*	Yes
	Dysphagia	0.34	0.52	0.08	< 0.001*	Yes
Gastrointestinal Distress /Sleepiness	Gastrointestinal Distress	0.73	0.57	0.09	< 0.001*	Yes
	Daytime Sleepiness	0.69	0.84	0.08	< 0.001*	Yes
	Chronic Fatigue	0.51	0.36	0.10	< 0.001*	Yes
	Sleep Apnea	0.47	0.78	0.09	< 0.001*	Yes
	Hand Stiffness	0.36	0.29	0.10	0.005*	Yes

*Note:* CFA model was modified by removing the non-significant signs and symptoms. CFA correlations are 0.60 between Factors Skeletal Muscle Weakness and Facial Weakness/Myotonia; 0.57 between Factors Skeletal Muscle Weakness and Gastrointestinal Distress/Sleepiness; and 0.43 between Factors Facial Weakness/Myotonia and Gastrointestinal Distress/Sleepiness. ^*^ indicates statistically significant *p* < 0.05. SE = standard error

Positive associations were detected between CTG repeat length and factor scores. For all three factors, those with higher CTG repeat length have higher scores. Being male, having an older age at onset, and having longer follow-up years are associated with higher score of skeletal muscle weakness. An older age at onset is also associated with higher score of gastrointestinal distress /sleepiness. No statistically significant associations were found between family history and race/ethnicity (Table 4).

**Table 4:** Associations of covariates with factor scores, among adult-onset DM1 participants from MD STAR*net* (*N* = 115 ^a^)

Covariates	Facial Weakness/Myotonia score					Skeletal Muscle Weakness score					Gastrointestinal Distress /Sleepiness score				
	Estimate	SE	DF	t	*p*	Estimate	SE	DF	t	*p*	Estimate	SE	DF	t	*p*
**Intercept**	-1.4	0.42	5	-3.50	0.017	-2.20	0.38	5	-5.72	0.002	-1.25	0.31	5	-3.99	0.010
**Log (CTG repeat length)**	0.17	0.06	103	2.98	0.004*	0.21	0.05	103	3.91	< 0.001*	0.13	0.04	103	3.00	0.003*
**Age of onset**	0.001	0.003	103	0.43	0.668	0.01	0.003	103	4.22	< 0.001*	0.01	0.002	103	2.38	0.019*
**Sex**															
Female	Reference group					Reference group					Reference group				
Male	0.21	0.10	103	2.19	0.031	0.24	0.09	103	2.79	0.006*	0.08	0.07	103	1.15	0.254
**Race/ethnicity**															
Other	Reference group					Reference group					Reference group				
Non-Hispanic White	0.11	0.11	103	0.99	0.326	0.14	0.10	103	1.37	0.173	0.13	0.08	103	1.61	0.111
**Family History**															
Yes	Reference group					Reference group					Reference group				
No	-0.02	0.13	103	-0.18	0.861	0.18	0.12	103	1.57	0.120	-0.02	0.10	103	-0.26	0.796
**Follow-up years**	0.01	0.01	103	2.24	0.028	0.03	0.006	103	4.19	< 0.001*	0.01	0.005	103	2.24	0.027

*Note*^a^: Among the 228 patients, there are missing values for age of onset, age at genetic testing, and family history. The sample size for the regression analysis is 115. The outcome is factor score, the independent variables are the logarithm CTG repeat length, age of onset, sex, race/ethnicity, family history, years of follow-up since onset, and site (which is fit as a random effect). * indicates p-value of covariates < adjusted significance threshold. Benjamini-Hochberg procedure is used to control the false discovery rate

## Discussion

In this cross-sectional study, we identified three latent factors of S/S that develop during the clinical course of adult-onset DM1. Many of our findings confirm previous reports, including the finding that there was 6.5 years between the first record of reported S/S to genetic testing for our study group, which is consistent with two published studies [11, 29].

Previous studies reported that approximately 90% of patients with DM1 have muscle weakness, problems with hands or arms, fatigue, myotonia, and impaired sleep or daytime sleepiness [11, 29, 30]. Considering that different terms for symptoms were used in the surveilled medical records (e.g., muscle weakness could involve facial weakness, distal weakness, hand weakness and proximal weakness), our findings are comparable with these previous studies. However, fatigue and myalgia were recorded less often in this study, with only 37% of DM1 patients noted fatigue and 20.2% noted pain in their medical records. A prospective study in DM1 patients found that fatigue and daytime sleepiness increased over 9 year period and were modulated by CTG repeats [19]. The lower percentage of DM1 patients reporting fatigue in our study could be influenced by shorter follow-up lengths in MD STAR*net*. Different from registry or prospective cohort studies, our study utilized population-based data, which involves the unbiased inclusion of participants and the unbiased data on clinical care approach taken by clinicians during ordinary health care. However, the study is also subject to the different practice styles of individual clinicians outside of an overarching prespecified data collection approach.

Factor analysis provided us a unique perspective to identify symptoms that tend to co-occur in the clinical course. Outside of survey-based studies [11, 30], most longitudinal observational studies report on only one body system at a time. Interestingly, gastrointestinal distress, chronic fatigue, sleep disorder and hand stiffness were grouped together. In the general adult population, excessive daytime sleepiness has been significantly associated with gastrointestinal issue such as ulcer-like dyspepsia, irritable bowel syndrome, functional constipation, and gastroesophageal reflux disease [31]. Additionally, proinflammatory cytokines play a role in sleep dysfunction and are altered in gastrointestinal diseases [32]. In adult-onset DM1 patients, there is a positive correlation between fatigue levels and excessive daytime sleepiness and suggests that fatigue and excess daytime sleepiness should be evaluated together since both have a great impact on patients’ quality of life [33]. Based on our current knowledge, there is a lack of literature regarding the high correlation between sleep disorders, fatigue, and gastrointestinal distress among DM1 patients; therefore, our findings merit replication.

It is noteworthy that some S/S, which clinicians would typically group together, were separated using factor analysis. For example, in our data, hand stiffness loaded higher into the Gastrointestinal Distress/ Sleepiness (Group 3) instead of skeletal muscle weakness (Group 2), meaning patients who report hand stiffness are also highly likely, at some stage of their disease progression, to report gastrointestinal disturbances and sleep problems. One explanation may be that physicians think in terms of presenting S/S and impacted organ systems or functions, rather than the cumulative occurrence of S/S over the clinical course. Our approach used cumulative S/S which yielded some novel insights. Another explanation may be that hand stiffness represents some gradient of myotonia, and that people with S/S in gastrointestinal disturbances and sleep problems are more likely to report having hand stiffness than full-fledge myotonia [34]. Note that validation of the factor analysis can strengthen our study. Due to the limited sample size, a cross-validation approach was not applied. Instead, we used the goodness of fit statistics as an indirect indication of the validity, and the results show that three factors are acceptable (Supplementary Table 1). For external validation, our findings should be confirmed in prospective longitudinal studies. It also suggests a need for further research to better understand correlation patterns of S/S, as well as the pathophysiological mechanism. While many of these patients did have overlapping S/S, factor analyses suggest which clinical features are most tightly linked. This may be important for longitudinal management.

CTG repeat length is known to be associated with the onset and severity of DM1. Our results substantiate this finding since those with higher CTG repeats have higher S/S scores. We also found that individuals with later age of onset tend to report more S/S related to skeletal muscle weakness and gastrointestinal distress /sleepiness. In terms of sex differences, males have a higher score of skeletal muscle weakness compared to females. However, we didn’t observe a significant sex difference in GI disorders, as reported in the literature [10, 35]. Unlike other studies focusing only on GI disorders, our results using factor score that combines GI distress with sleep disorders, chronic fatigue, and hand stiffness.

We didn’t find statistically significant difference in factor scores between non-Hispanic White individuals and others. There is limited literature on the variation in S/S across racial/ethnic groups, but studies have shown different CTG repeats by race/ethnicity. For example, a study conducted in Alabama reported African Americans had lower CTG repeats compared to other race/ethnic groups [36]. In our study, 71.9% of the population was non-Hispanic White. Further replication in studies powered to detect differences between racial/ethnic groups is needed.

There are limitations to this study. Surveillance was limited to six U.S. geographic areas, thus the surveilled group are not necessarily representative of all people with DM1. Surveillance data are different from longitudinal study data since we could only capture recordings on the clinical records that were available when surveillance was conducted. The use of surveillance data is an important addition to research data since it is population based, and thus it provides an accurate representation of what is recorded during clinical visits in a surveilled area. There is a wide literature using MD STAR*net* surveillance data to describe many muscular dystrophies, including DM1 [25, 37–43]. Second, surveillance was conducted primarily at neuromuscular clinics; thus, it’s possible that individuals who received diagnostic and treatment services in primary care settings were not included in MD STAR*net*. Third, bias could be introduced by different follow-up lengths (e.g., a recently diagnosed DM1 individual would have a shorter follow-up period compared to those diagnosed earlier, potentially leading to fewer observed S/S) and missing values from medical records (e.g., age at first symptom). In addition, data from a surveillance program is constrained by the involvement of multiple providers who use various electronic medical record systems, and document medical history and progress notes in different approaches. Finally, since DM1 is a multisystem disease with a highly variable presentation among individuals, our findings did not include all S/S. For instance, neurocognitive symptoms (e.g., apathy, executive cognitive functioning, social functioning, depression), endocrine symptoms, cardiac and urogenital symptoms are not captured. S/S related to developmental delay and respiratory failure/insufficiency were not reported because surveillance data must exclude conditions affecting less than 11 individuals. Further study with larger sample size should be conducted to provide a full disease spectrum and to identify the mechanisms that contribute to the identified latent factors.

Despite these limitations, our study used data collected from a population-based surveillance system and identified cases at neuromuscular clinics and other medical sites within the six catchment areas. This reduced the possibility of bias in case selection. Further, we included a geographically, ethnically, and racially diverse surveilled population.

## Conclusions

This is a large population-based study from six U.S. sites with geographic, ethnic, and racial diversity in the surveilled population. The identified latent factors of S/S offer insights for clinicians to consider in the perspective of the clinical course.

## Electronic supplementary material

Below is the link to the electronic supplementary material.

Supplementary Material 1

## Data Availability

Data from each site were pooled at the MD STAR*net* Coordinating Center without names or addresses, but still containing sensitive information. Data from the MD STAR*net* are not publicly available (researchers interested in MD STAR*net* may contact MD STARnet@cdc.gov).

## Abbreviations

CDC: Centers for Disease Control and Prevention
CFA: Confirmatory factor analysis
DM1: Myotonic dystrophy type 1
DMPK: Dystrophia myotonic-protein kinase gene
EFA: Exploratory factor analysis
ICD-9-CM: International Classification of Diseases, Ninth Revision, Clinical Modification
ICD-10-CM: International Classification of Diseases, Tenth Revision, Clinical Modification
IQR: Interquartile range
MD STAR*net*: Muscular Dystrophy Surveillance, Tracking, and Research Network
SD: Standard deviation
S/S: Signs and symptoms
