# Supplementary material for: Latent factors underlying the symptoms of adult-onset myotonic dystrophy type 1 during the clinical course
Source: Orphanet J Rare Dis. 2024 Nov 1;19:409. doi: 10.1186/s13023-024-03359-8 (PMC11529289; doi:10.1186/s13023-024-03359-8)
Supplement: Supplementary file 1 — Supplementary Material 1 [file 13023_2024_3359_MOESM1_ESM.docx]

**Supplementary Table 1. Fit statistics from Exploratory Factor Analysis (EFA) and Confirmatory Factor Analysis (CFA), among adult-onset DM1 participants in MD STAR*net***

| **Fit statistics** | **EFA** | | | **CFA** | **General Goodness of Fit [1]** |
| --- | --- | --- | --- | --- | --- |
|  | **1 factor** | **2 factors** | **3 factors** | **3 factors** |  |
| RMSEA  (Root mean square error of approximation) | 0.049 | 0.041 | 0.027 | 0.029 |  |
| RMSEA 90% CI | (0.036, 0.062) | (0.024, 0.056) | (0.000, 0.046) | (0.000,0.048) |  |
| Probability RMSEA <=0.05 | 0.520 | 0.829 | 0.983 | 0.966 | Good: p > 0.05 |
| CFI (comparative fit index) | 0.835 | 0.899 | 0.963 | 0.965 | Good: CFI or TLI > 0.95 Acceptable: CFI or TLI >0.90 |
| TLI (Tucker–Lewis index) | 0.813 | 0.869 | 0.944 | 0.958 |  |
| SRMR  (Standardized root mean square residual) | 0.130 | 0.113 | 0.088 | 0.084 | Good: SRMR<0.08 Acceptable: SRMR<0.10 |

Note: EFA model with three groups have acceptable goodness of fit. EFA with three groups were selected after consultation with clinician experts.

[1] Hu Lt, Bentler PM. Cutoff criteria for fit indexes in covariance structure analysis: Conventional criteria versus new alternatives. Structural equation modeling: a multidisciplinary journal. 1999;6(1):1-55.

**Supplementary Table 2. Grouping of Signs and symptoms (S/S) among adult-onset DM1 participants in MD STAR*net***

| **Grouped S/S** | **S/S before grouping** |
| --- | --- |
| Developmental Delays | Developmental Delay |
|  | Global Developmental Delay |
|  | Globally Delay |
|  | Gross Motor Delay |
|  | Delayed Development and Short Stature |
|  | Decreased Movement/Fixed Extension of Ankles/polyhydramnios |
|  | Low Tone and Motor Delays |
|  | Plagiocephaly |
| Gastrointestinal Distress | Diarrhea/Abdominal Pain/Constipation |
|  | Irritable Bowel Syndrome |
| Daytime Sleepiness | Narcolepsy |
|  | Daytime Sleepiness |
| Myalgia | Back and Leg Pain |
|  | Leg Pain |
|  | Ankle and Knee Pain |
|  | Buttock and Bilateral Leg Pain |
|  | Chronic Back Pain that Radiates to The L Thigh |
|  | Generalized Pain and Weakness |
|  | Knee Pain and Swelling |
|  | Lower Extremity Pain |
|  | Muscle Pain |
|  | Pain and Clumsiness |
|  | Pain in Lower Extremities |
|  | Pain in Thighs, Back and Neck |
|  | Paresthesia of Legs |
|  | Rhabdomyolysis/Severe Muscle Cramps |
|  | Ache in Muscles |
|  | Muscle Cramp |
|  | Fibromyalgia, Chronic Pain Syndrome |
|  | Muscle Pain |
|  | Nerve Pain |
|  | Neuropathy |
